# Altered gut microbiota composition with antibiotic treatment impairs functional recovery after traumatic peripheral nerve crush injury in mice: effects of probiotics with butyrate producing bacteria

**DOI:** 10.1186/s13104-022-05967-8

**Published:** 2022-02-23

**Authors:** Andrew Rodenhouse, M. A. Hassan Talukder, Jung Il Lee, Prem Kumar Govindappa, Mary O’Brien, Kristen M. Manto, Kelsey Lloyd, Grant D. Wandling, Justin R. Wright, Jeremy R. Chen See, Samantha L. Anderson, Regina Lamendella, John P. Hegarty, John C. Elfar

**Affiliations:** 1 Department of Orthopaedics and Rehabilitation, Center for Orthopaedic Research and Translational Science, The Pennsylvania State University College of Medicine, Milton S. Hershey Medical Center, Mail code H089, 500 University Drive, P.O. Box-850, Hershey, PA USA; 2 Department of Orthopedic Surgery, Korea University Guro Hospital, Seoul, South Korea; 3 Wright Labs LLC, Huntingdon, PA USA; 4 Juniata College, Huntingdon, PA USA

**Keywords:** Antibiotics, Probiotics, 16S rRNA analysis, Gut microbiota, Peripheral nerve injury, Functional recovery

## Abstract

**Objective:**

Antibiotics (ABX) are widely used for life-threatening infections and also for routine surgical operations. Compelling evidence suggests that ABX-induced alterations of gut microbiota composition, termed dysbiosis, are linked with diverse disease states including neurological and neurodegenerative conditions. To combat the consequences of dysbiosis, probiotics (PBX) are widely used. ABX-induced dysbiosis is reported to impair neurological function after spinal cord injury. Traumatic peripheral nerve injury (TPNI) results in profound neurologic impairment and permanent disability. It is unknown whether ABX treatment-induced dysbiosis has any impact on TPNI-induced functional recovery, and if so, what role medical-grade PBX could have on TPNI recovery.

**Results:**

In this study, ABX-induced dysbiosis and PBX-induced microbiota enrichment models were used to explore the potential role of gut microbiome in TPNI. Stool analysis with 16S ribosomal RNA (rRNA) gene sequencing confirmed ABX-induced dysbiosis and revealed that ABX-induced changes could be partially restored by PBX administration with an abundance of butyrate producing bacteria. Pre-injury ABX significantly impaired, but pre-injury PBX significantly improved post-TPNI functional recovery. Importantly, post-injury PBX protected against pre-injury ABX-induced functional impairment. These findings demonstrate that reestablishment of gut microbiota composition with butyrate producing PBX during ABX-induced dysbiosis could be a useful adjuvant therapy for TPNI.

**Supplementary Information:**

The online version contains supplementary material available at 10.1186/s13104-022-05967-8.

## Introduction

The gut microbiota play an important role in normal host physiology and health [1–3]. Alteration of the host resident intestinal microbiome, termed dysbiosis, has been implicated in many disease states including gastrointestinal, metabolic, autoimmune, inflammatory, neuropsychiatric, and neurodegenerative disorders [1–8]. To combat the consequences of dysbiosis, nutritional interventions, consisting of probiotics (PBX), are widely used [9–13]. PBX create a healthy gut environment by downregulating pathogenic bacteria in favor of other more beneficial bacterial populations.

Multiple factors including age, genetics, environmental stress, infection, diet, and antibiotics (ABX) can contribute to dysbiosis [11, 14]. Antibiotics are widely used in medicine and surgery [15–17]. Although ABX are essential for the prevention and treatment of bacterial infections and have significantly improved treatment outcomes, as many as 5–30% of people who receive ABX suffer from adverse effects [17–20]. Besides common side effects, several studies have shown that ABX treatment results in short- or long-term changes in the intestinal microorganisms (microbiota) in both humans and animals [21–26].

Gut bacteria produce a wide range of biologically active molecules, such as metabolites, short-chain fatty acids (SCFAs), proteins and enzymes. SCFAs such as, acetic, propionic and butyric acids, are some of the most important gut microbial products, and they are involved in a range of regulatory activities beneficial to the host [1, 8, 27, 28]. For example, butyrate has both intestinal and systemic anti-inflammatory, pro-inflammatory, immunomodulatory, and anti-oxidant effects [27–30]. ABX pretreatment has been shown to cause dysbiosis in mice with significant effects in normal health and disease conditions [31–33]. A deficiency of gut microbiota in mice affects the distribution and maturation of microglia and impairs the innate immune responses in the brain [34]. Dysbiosis caused by ABX has been shown to impair corneal nerve regeneration in mice by affecting macrophage distribution [32]. ABX-induced dysbiosis is also reported to impair the recovery of neurological function in mice after traumatic spinal cord injury, whereas medical-grade PBX treatment improves recovery [31]. Traumatic peripheral nerve injury (TPNI) causes profound neurologic impairment and permanent disability [35], and inflammatory responses occurring after TPNI play a critical role in nerve regeneration and functional recovery [36, 37]. Although ABX are routinely used in traumatic neuromuscular injuries, it is unknown whether ABX-induced dysbiosis has similar impacts on TPNI-induced functional recovery as reported in spinal cord injury. While the beneficial anti-inflammatory effects of SCFAs extend beyond the gut [8, 27, 28], nothing is known regarding the role of medical-grade PBX containing butyrate-producing bacteria in TPNI recovery where inflammation plays a critical role in TPNI repair and functional recovery.

It is not ethical and possible to do experimental nerve injury study in humans. Therefore, in this study, using both loss-of-function (ABX-induced dysbiosis) and gain-of-function (PBX-induced microbiota enrichment) microbiome models, we characterized the gut microbiome in mouse stools and explored the potential role of the gut microbiome composition in the functional recovery of TPNI.

## Main text

### Materials and methods

#### Animals

The experimental procedures were reviewed and approved by the Institutional Animal Care and Use Committee (IACUC) at the Pennsylvania State College of Medicine and the experiments were performed according to the guidelines of IACUC. A total of 44 10-week-old male C57BL/6J mice (Jackson Laboratories, Bar Harbor, Maine, USA) weighing 20–25 g were used for the study. Animals were housed and routinely monitored at the animal facility according to IACUC guidelines.

#### Antibiotic cocktail and probiotics treatments, experimental groups, stool sample collection and analyses

The terms “ABX” and “ABX cocktail” are interchangeably used in this manuscript to denote the effects of antibiotics. An ABX cocktail consisting of 2 g/L streptomycin, 0.17 g/L gentamicin, 0.125 mg/L ciprofloxacin, and 1 g/L bacitracin was prepared in drinking water [31], and VSL#3 (Sigma-Tau Pharmaceuticals) was suspended in sterile saline (5 billion bacteria in 400 µL saline) [31]. The experimental groups, stool sample collection, stool 16S ribosomal RNA (rRNA) gene sequencing DNA extraction and analysis [38], bioinformatics analysis [39–43], overall community composition analysis [44], alpha diversity analysis [45–47], beta diversity analysis [48, 49], and biomarker analysis [50] are described in details in Additional file 1: Methods and materials and Additional file 2: Fig. S1.

#### Mouse model of severe sciatic nerve crush injury and functional analysis

An established severe sciatic nerve crush injury model was utilized [51, 52] and functional analysis was performed before and after different treatments [51, 53, 54] as described in Additional file 1.

#### Data analysis

All results are presented as means ± SEM. Functional data were analyzed by a mixed model 2-way ANOVA for multiple comparisons with Tukey’s correction using the GraphPad PRISM 8 (GraphPad Software, San Diego, CA, USA). Significant differences for microbiome alpha diversity between the groups were assessed using Kruskal–Wallis tests through QIIME2. Likewise, beta diversity differences were assessed using PERMANOVA tests through QIIME2. Wilcoxon Rank Sum tests within R were used to test for significant differences among the most abundant phyla. A *P* value of < 0.05 was considered a statistically significant value.

### Results

#### Effects of ABX and PBX on gut microbiota composition

**Fig. 1:**
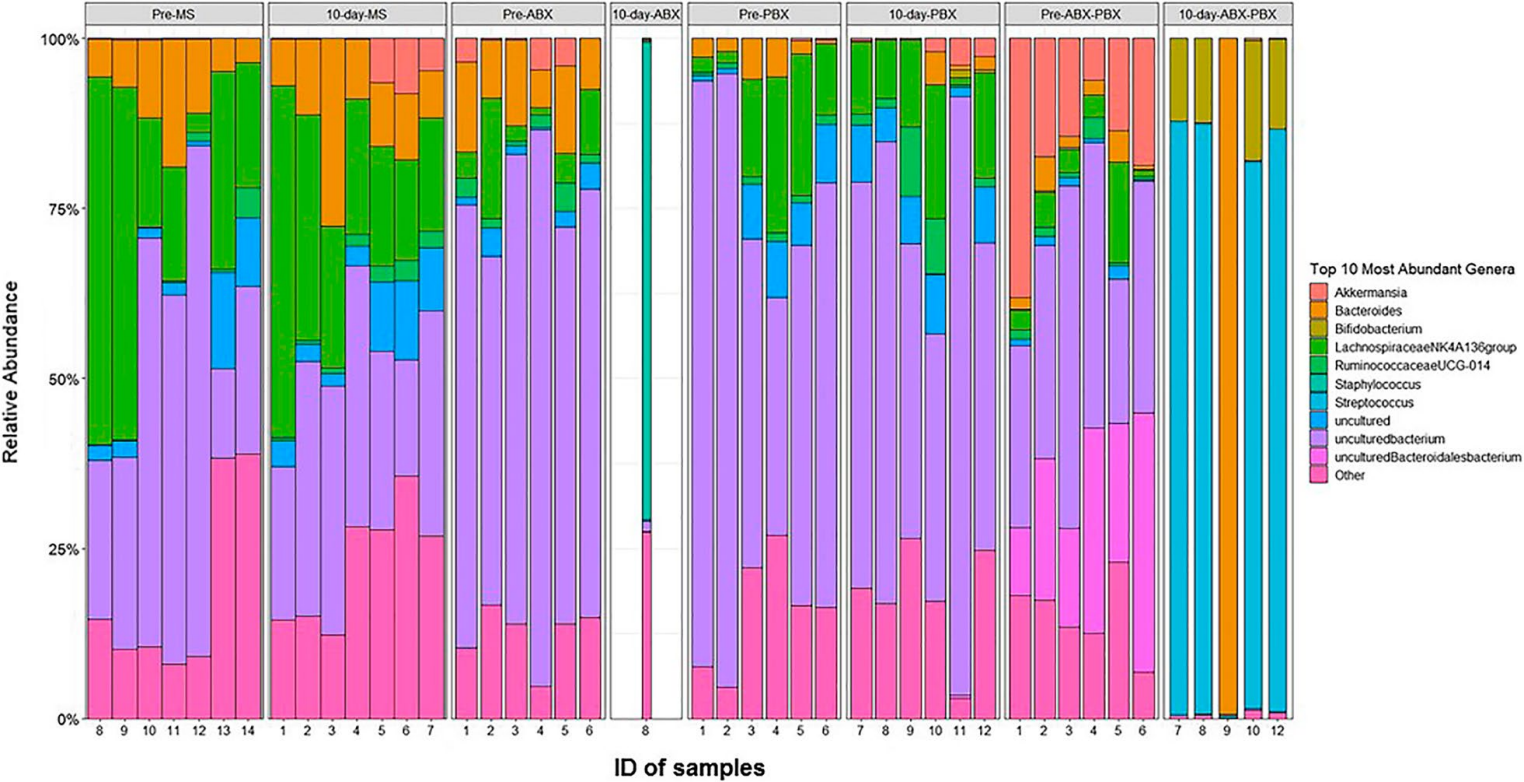
Microbiota dynamics at the genus level. The relative abundance of the most prevalent taxa in control (Pre-) and respective 10-day-vehicle (MS, maple syrup in drinking water), ABX, PBX, and ABX-PBX stool samples of mice. Bar plot is made with the ggplot2 package in R. Genera are shown in different colors. Number at the bottom denotes the ID number of each sample

Figure 1 shows the relevant abundance of the most prevalent genera within all groups after fecal 16S rRNA sequencing and analysis. Compared to other groups, only one of six 10-day ABX samples yielded enough sequences to be included in analysis, and it had a very distinct compositional profile, being dominated by *Staphyloccocus*. Among the other groups, additional differences are evident, such as the increased abundance of *Streptococcus* in the 10-day-ABX-PBX group. These differences clearly demonstrate the impact of various treatment protocols on gut bacterial communities.

Additional file 3: Table S1 shows the top six characteristic bacteria at the phylum level of each group before and after treatments using 16S rRNA sequencing. The gut microbiota was absent in most fecal samples after ABX treatment and only one fecal sample in 10-day-ABX group yielded enough sequences to be characterized. At the phylum level, the ABX treatment resulted in a significant increase in *Firmicutes* and a sharp decline in *Bacteroidetes* compared with the Pre-ABX group. Furthermore, ABX treatment also led to an increase in *Proteobacteria* and *Actinobacteria*. While bacterial taxa in PBX group samples remained stable compared to Pre-PBX group, PBX treatment blunted the effect of ABX in ABX-PBX group with an increase in *Firmicutes* and *Actinobacteria* strains and a decrease in *Bacteroidetes* strain compared to Pre-ABX-PBX group.

#### Alpha-diversity and beta-diversity analyses of gut microbiota

**Fig. 2:**
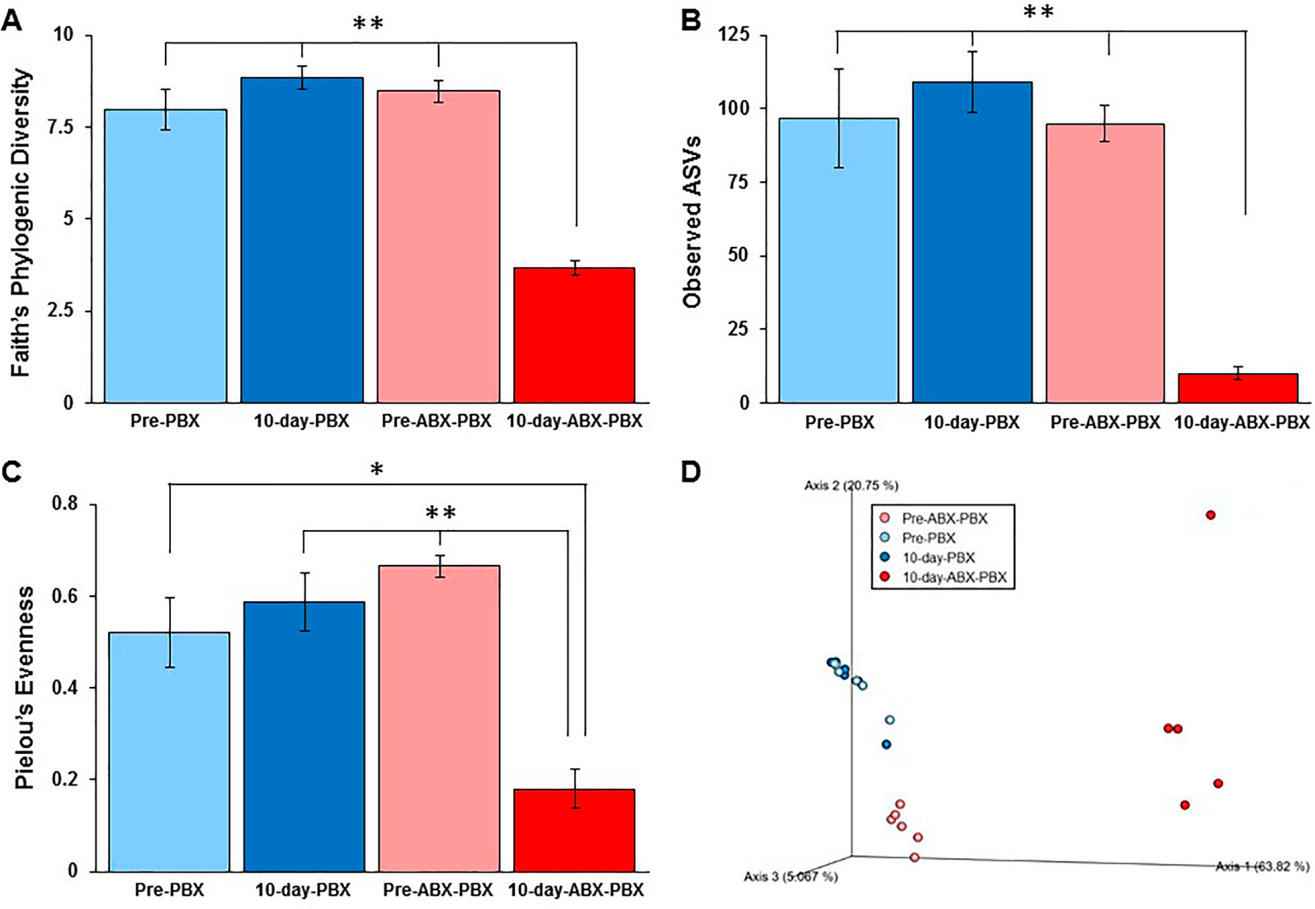
Alpha diversity and beta diversity of Pre-PBX, 10-day-PBX, Pre-ABX-PBX, and 10-day-ABX-PBX stool samples of mice (n = 5–6/group). All diversity analyses were performed in QIIME2. Within-sample diversities were measured by Faith’s phylogenic diversity (**A**), **P < 0.01, 10-day-ABX-PBX vs. Pre ABX-PBX, Pre-PBX, and 10-day-PBX groups; observed amplicon sequence variants (ASVs) (**B**), **P < 0.01, 10-day-ABX-PBX vs. Pre ABX-PBX, Pre-PBX, and 10-day-PBX groups; and Pielou’s evenness (**C**), **P < 0.01, 10-day-ABX-PBX vs. Pre ABX-PBX and 10-day-PBX groups, *P < 0.05, 10-day-ABX-PBX vs. Pre-PBX group. Between-sample dissimilarities were measured by Principal Coordinates Analysis (PCoA) based on weighted UniFrac distances (**D**), and the clustering of 10-day-ABX-PBX group was significantly different (**P < 0.01) from Pre ABX-PBX, 10-day-PBX and Pre-PBX groups

Bacterial richness within each fecal sample was determined using three different alpha diversity methods: Faith’s phylogenetic diversity, observed amplicon sequence variants (ASVs), and Pielou’s evenness. The observed alpha-diversity values in 10-day-ABX-PBX group were significantly lower compared with other groups as shown in Fig. 2 as Fig. 2A (**P < 0.01), Fig. 2B (**P < 0.01), and Fig. 2C (*P < 0.05, **P < 0.01), respectively.

Beta diversity of the fecal samples was calculated using the weighted UniFrac distances (Fig. 2D). The principal coordinates analysis (PCoA) demonstrated that Pre-PBX and 10-day-PBX samples (dots) cluster closely together on the plot and were not significantly different. In contrast, the 10-day-ABX-PBX samples clustered differently and away from all groups.

#### Microbiota biomarkers and taxonomic plots analyses

The linear discriminant analysis effect size (LEfSe) test and cladogram plot from LEfSe analysis were used to identify the taxa that had significantly different abundances within the same treatment group or different treatment groups. As shown in Additional file 4: Fig. S2, a significant abundance of *Akkermansia* (Additional file 4: Fig. S2B) in 10-day-PBX samples compared to Pre-PBX samples (*P* < 0.05), a significant abundance of *Bifidobacteriales* (Additional file 4: Fig. S2D) in 10-day-ABX-PBX samples compared to Pre-ABX-PBX samples (*P* < 0.05), and a greater abundance of *Lactobacillales* (Additional file 4: Fig. S2F) in 10-day-ABX-PBX samples compared to 10-day-PBX samples (*P* = 0.051) were observed.

#### Effect of ABX and PBX-treatments on the functional recovery after TPNI

**Fig. 3:**
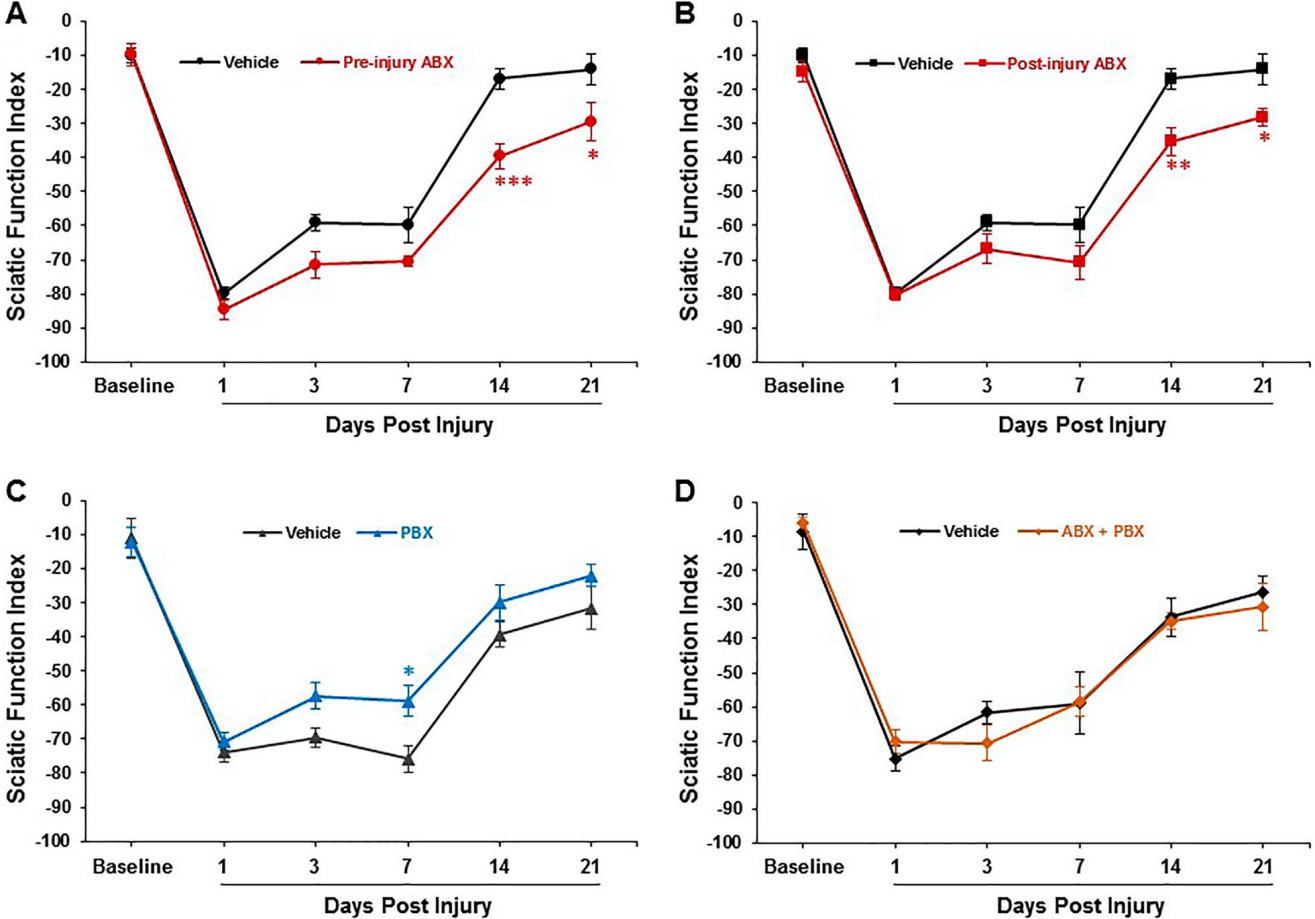
Functional recovery as sciatic function index (SFI) from baseline in vehicle, Pre-ABX, Post-ABX, Pre-PBX, and ABX-PBX groups. ABX-induced dysbiosis impairs SFI (**A**, **B**), whereas PBX accelerates SFI recovery (**C**) and protects against ABX-induced SFI deterioration (**D**). Data are expressed as the mean ± SEM, *p < 0.05, **P < 0.01, ***P < 0.001, vehicle vs. respective Pre-ABX, Post-ABX, and Pre-PBX group, n = 5–7/group

Sciatic function index (SFI) is the primary functional outcome measure after TPNI. We observed that that pre-injury ABX treatment significantly impaired SFI recovery after crush injury compared the vehicle group (Fig. 3A). To determine whether SFI recovery is dependent on dysbiosis timing, a post-injury ABX group was tested. Similar to the Pre-ABX group, the Post-ABX group also demonstrated significantly impaired functional recovery compared to vehicle (Fig. 3B). In contrast, mice receiving daily PBX (VSL#3) demonstrated significantly improved SFI recovery (Fig. 3C). To further investigate whether or not PBX could rescue the post-injury functional deficits observed following pre-injury ABX administration, a pre-injury ABX plus post-injury PBX group was investigated. Figure 3D shows that PBX prevented any substantial functional deficits in the treatment group when compared to vehicle group.

### Discussion

The main findings of this study in loss-of-function (ABX treatment) and gain-of-function (PBX treatment) models of gut microbiota are: ABX impair the functional recovery after peripheral nerve injury, and PBX improve or rescue post-injury functional recovery in the absence or presence of ABX, respectively. These findings, coupled with the observation of ABX-induced drastic depletion of the gut microbiota community with lower diversity and PBX-induced restoration of gut microbiota community with an increased abundance of butyrate producing bacteria after ABX-induced depletion of host microbiota, suggest an important role of the gut microbiome modulation in the functional recovery of TPNI.

ABX are often used in many clinical scenarios, including infection prevention for trauma and surgical patients [55–57]. ABX act not only at their intended sites, but also in other distant tissues. PBX are defined as live microorganisms conferring a health benefit on the host when administered in adequate amounts [58], and VSL#3 is a medical grade probiotic mixture that contains 8 different strains of “good” bacteria within the orders *Lactobacillales* or *Bifidobacteriales* [9, 59, 60]. In this study, 16S rRNA sequencing identified significant compositional changes that occur in the gut microbiome secondary to ABX or PBX administration and these findings are consistent with previously published findings in mice with ABX or PBX [33]. A markedly separated distribution (beta diversity) of microbiota confirmed that the various treatments created unique bacterial communities within each group as evidenced by the distinct clustering patterns visualized on the PCoA plot. While 10-day-ABX mice failed to retain enough microbiome for analysis, 10-day-ABX-PBX mice were able to reestablish a modest amount of the intestinal microbiome. The taxa enriched in the 10-day-ABX-PBX group fell within the orders *Lactobacillales* and *Bifidobacteriales*. Taken together, these findings confirm the gut microbiota reestablishing effect of VSL#3 against ABX-induced dysbiosis.

Importantly, the accelerated and rescued functional recovery with VSL#3 after TPNI provides direct evidence for a functional link between the gut microbiome and TPNI recovery. VSL#3 is reported to prevent the host from stable pathologic colonization in different experimental models [9, 59, 60]. In a mouse model of spinal cord injury, VSL#3 conferred neuroprotection with improved locomotor recovery [31]. Supplementation with VSL#3 is also reported to rescue hippocampal neurogenesis and brain function in ABX-treated mice [61]. Recently, ABX-induced dysbiosis was shown to impair corneal nerve regeneration in mice, an effect that was largely reversed by VSL#3 treatment [32]. We found an increased abundance of butyrate-producing bacteria *Ruminococcaceae* in the 10-day-PBX group and *Bifidobacterium* in the 10-day-ABX-PBX group. In addition, *Akkermansia* was significantly abundant in 10-day-PBX group, and it is reported to produce butyrate, propionate and acetate [29]. Since the beneficial anti-inflammatory effects of SCFAs extend beyond the gut and macrophages are critical for the inflammatory response after TPNI, it is thus possible that anti-inflammatory and immunomodulatory effects of SCFAs-producing gut microbiota after VSL# 3 treatment could be involved in our proof-of-concept study.

In conclusion, our study provides direct evidence for an important role of the gut microbiome in the functional recovery after sciatic nerve crush injury. We demonstrate that ABX-induced dysbiosis impairs TPNI-induced functional recovery, pre-injury PBX treatment promotes functional recovery, and most interestingly, PBX can effectively “rescue” ABX-treated mice from the functional consequences of ABX-induced dysbiosis.

## Limitations

Our study has some limitations: *First*, we performed analysis on fecal microbiota abundance, diversity and biomarkers, but not on the enriched microbial-derived metabolites or neurochemicals. *Second*, we did not investigate the time-dependent molecular and cellular changes in the injured nerve with or without ABX, PBX, and ABX plus PBX treatments*. Third*, it is unknown if ABX or PBX treatments would have any effect on nerve myelination and conduction velocity.

## Supplementary Information

**Additional file 1.** Materials and methods: detailed experimental protocol of the study, including animals, ABX and PBX administrations, stool sample collection, stool 16S rRNA analysis, bioinformatics analysis, overall community composition analysis, alpha diversity analysis, beta diversity analysis, and biomarker analysis.

**Additional file 2: Figure S1.** Experimental groups and time lines. Vehicle for ABX group received autoclaved drinking water supplemented with maple syrup daily beginning 7 days before nerve injury (Day − 7); Pre-injury ABX group received the antibiotic cocktail in drinking water daily beginning 7 days before nerve injury (Day − 7); Post-injury ABX group received the antibiotic cocktail in drinking water daily immediately after nerve injury (Day 0); Vehicle for PBX group received 400 µL sterile saline via oral gavage daily beginning 7 days before nerve injury (Day − 7); Pre-injury PBX group received probiotics suspension via oral gavage daily beginning 7 days before nerve injury (Day − 7); Vehicle for ABX and PBX group received autoclaved drinking water supplemented with maple syrup daily beginning 7 days before nerve injury (Day − 7) plus 400 µL sterile saline via oral gavage daily immediately after nerve injury (Day 0); and sequential ABX + PBX group received the antibiotic cocktail in drinking water daily beginning 7 days before nerve injury (Day − 7) plus probiotics suspension via oral gavage immediately after nerve injury (Day 0). All groups received a similar crush injury and each treatment regimen was continued daily until the end of protocol at day 21. Functional analysis as sciatic function index (SFI) and fecal sampling were performed at indicated days.

**Additional file 3: Table S1.** The top characterized taxa at the phylum level of each group before (Pre-) treatment and at 10-day of the study.

**Additional file 4: Figure S2.** Taxonomic differences of fecal microbiota between different groups of mice. **A** Cladogram using LEfSe method showing the phylogenetic relationships among the enriched taxa within Pre-PBX (light blue) and 10-day-PBX groups (blue). **B** The relative abundance of *Akkermansia* was significantly higher in 10-day-PBX group. *P < 0.05, n = 6/group. **C** Cladogram using LEfSe method showing the phylogenetic relationships among the enriched taxa within Pre-ABX-PBX (pink) and 10-day-ABX-PBX (red) groups. **D** The relative abundance of *Bifidobacteriales* was significantly higher in 10-day-ABX-PBX group. *P < 0.05, n = 5–6/group. **E** Cladogram using LEfSe method showing the phylogenetic relationships among the enriched taxa within 10-day-PBX (blue) and 10-day-ABX-PBX (red) groups. **F** The relative abundance of *Lactobacillales* was markedly higher in 10-day-ABX-PBX group. *P* = 0.051, n = 5–6/group.

## Data Availability

The datasets used and/or analyzed for this study are stored at secured institutional server and will be available from the corresponding author.

## Abbreviations

ABX: Antibiotics
AVSs: Amplicon sequence variants
IACUC: Institutional Animal Care and Use Committee
LEfSe: Linear discriminant analysis effect size
PBX: Probiotics
rRNA: Ribosomal RNA
SFI: Sciatic function index
SCFAs: Short-chain fatty acids
TPNI: Traumatic peripheral nerve injury
