# Supplementary material for: Altered gut microbiota composition with antibiotic treatment impairs functional recovery after traumatic peripheral nerve crush injury in mice: effects of probiotics with butyrate producing bacteria
Source: BMC Res Notes. 2022 Feb 23;15:80. doi: 10.1186/s13104-022-05967-8 (PMC8867741; doi:10.1186/s13104-022-05967-8)
Supplement: Supplementary file 1 — Additional file 1. Materials and methods: detailed experimental protocol of the study, including animals, ABX and PBX administrations, stool sample collection, stool 16S rRNA analysis, bioinformatics analysis, overall community composition analysis, alpha diversity analysis, beta diversity analysis, and biomarker analysis. [file 13104_2022_5967_MOESM1_ESM.docx]

**Materials and Methods**

**Animals**

The experimental procedures discussed were reviewed and approved by the Institutional Animal Care and Use Committee (IACUC) at the Pennsylvania State College of Medicine and Milton S. Hershey Medical Center and the experiments were performed according to the guidelines of IACUC. A total of 44 ten-week-old male C57BL/6J mice (Jackson Laboratories, Bar Harbor, Maine, USA) weighing 20-25g were used for the study. Animals were housed at the animal facility in individually ventilated cage (3-5 mice/cage) with a 12-hour light-dark cycle at 65°-75°F temperature and 40-60% humidity, and they had free access to normal chow diet and water. Animals were routinely monitored according to IACUC guidelines by the research team, animal technicians, and institutional veterinarians.

The IACUC ensures that animal research conducted at Penn State Hershey is ethical, scientifically justified, performed in accordance with the approved IACUC protocol and compliant with the Guide, all federal, state and institutional regulations, and the institution's assurance and accreditation. The Penn State College of Medicine Animal Welfare Assurance Number on file with the Office of Laboratory Animal Welfare (OLAW) NIH is D16-00024 (previously A3045-01). The Committee’s composition and responsibilities regarding animal research are mandated by the Animal Welfare Act (Public Law 99-198).

**Antibiotic cocktail and probiotics treatments, and experimental groups**

As previously reported by Kigerl et al. [31], an antibiotic cocktail (ABX) consisting of 2 g/L streptomycin, 0.17 g/L gentamicin, 0.125 mg/L ciprofloxacin, and 1 g/L bacitracin was prepared in drinking water in combination with maple syrup (to make the regimen palatable) and administered *ad libitum* to mice. The antibiotic cocktail solution in drinking water was renewed every 3 days. VSL#3 (Sigma-Tau Pharmaceuticals), a medical grade PBX mixture, was suspended in sterile saline (5 billion bacteria in 400 µl saline) fresh every day and administered via oral gavage [31].

Animals were randomly assigned to each group and the experimental groups as shown in **Fig. S1** were: (1) Vehicle for ABX group (n =7) received autoclaved drinking water supplemented with maple syrup daily beginning 7 days before nerve injury (Day -7), (2) Pre-injury ABX group (n =7) received the antibiotic cocktail in drinking water daily beginning 7 days before nerve injury (Day -7), (3) Post-injury ABX group (n =7) received the antibiotic cocktail in drinking water daily immediately after nerve injury (Day 0), (4) Vehicle for PBX group (n =6) received 400 µL sterile saline via oral gavage daily beginning 7 days before nerve injury (Day -7), (5) Pre-injury PBX group (n =6) received probiotics suspension via oral gavage daily beginning 7 days before nerve injury (Day -7), (6) Vehicle for ABX and PBX group (n =5) received autoclaved drinking water supplemented with maple syrup daily beginning 7 days before nerve injury (Day -7) plus 400 µL sterile saline via oral gavage daily immediately after nerve injury (Day 0), and (7) ABX+PBX group (n =6) received the antibiotic cocktail in drinking water daily beginning 7 days before nerve injury (Day -7) plus probiotics suspension via oral gavage daily immediately after nerve injury at Day 0. Mice were housed in cages according to their experimental treatment to prevent cross-contamination of gut bacteria between different treatment groups [31]. One vehicle for ABX group was used for both Pre-injury ABX and Post-injury ABX groups. All groups received a similar crush injury and each treatment regimen was continued daily until the end of protocol at day 21. Functional analysis and fecal sampling were performed at the indicated days in **Fig. S1**.

**Stool sample collections**

Fecal samples were collected before ABX or PBX treatment and on the selected days of functional analysis (**Fig. S1**) to confirm gut dysbiosis and to compare the gut microbiota composition between treatment groups. Stool samples were placed into 1.5 mL sterile tubes, snap-frozen in liquid nitrogen, and stored at -80°C until analysis at Wright Labs (Huntingdon, Pennsylvania, USA).

**16S rRNA analysis of fecal samples**

16S ribosomal RNA (rRNA) gene sequencing DNA extraction was performed using the DNeasy PowerSoil kit (Qiagen, Frederick, Maryland, USA) according to the manufacturer’s protocol and eluted using 50 uL of DNase/RNase free water. After extraction, samples were quantified using an Invitrogen Qubit 4 Fluorometer and 1X Qubit dsDNA High Sensitivity Assay Kit (ThermoFisher Scientific, Waltham, Massachusetts, USA). All 16S rRNA illumina-tag PCR reactions were performed on DNA extracts per the Earth Microbiome Project’s protocol [38]. PCR products were pooled and gel purified on a 2% agarose gel using the QIAquick Gel Purification Kit (Qiagen, Frederick, Maryland, USA). Before sequencing, the purified pool was quality checked using an Agilent 2100 BioAnalyzer and Agilent DNA High Sensitivity DNA kit (Agilent Technologies, Santa Clara, California, USA). The purified pool was stored at -20˚C and then sequenced by Wright Labs LLC (Huntingdon, Pennsylvania, USA) using an Illumina MiSeq v2 chemistry with paired-end 250 base pair reads.

**Bioinformatic analysis procedures**

Raw data were imported into Qiime2 for processing and analyses [39]. Initial quality in the form of Phred q scores was determined using Qiime2, while cumulative expected error for each position was determined with VSEARCH [40]. Within Qiime2’s implementation of the DADA2 pipeline, forward reads were truncated at a length of 235 base pairs and reverse were truncated at a length of 155 base pairs, with a maximum expected error of 0.5 used for both, forward and reverse reads were merged and chimeras were removed, with the remaining sequences being assigned to amplicon sequence variants (ASVs).

Representative sequences were used to determine taxonomic information for the ASVs, using a Naive Bayes classifier in Qiime2, with a pre-trained Silva 132 database containing 515F/806R sequences [41]. Representative sequences were also used to create a rooted phylogenetic tree using MAFFT [42] and FastTree [43] through Qiime 2.

ASVs identified as Mitochondria or Chloroplasts were removed on the basis that they likely represented eukaryotic contamination. Samples with fewer than 1,500 sequences remaining after that filtration were removed from the ASV table.

**Overall community composition analysis**

The table was collapsed to the phylum level, and counts were converted to relative abundances. A table of averages was then created of the most abundant phyla, with abundances grouped by treatment (ABX, ABX-PBX, Maple Syrup (MS), PBX) and timepoint (Pre and 10-day of study). The “Pre” timepoint represented baseline samples obtained prior to any intervention. The 10-day of study timepoint is synonymous with 3 days post injury or day 10 of the study period. Only phyla that were among the three most abundant for at least one of those groupings were included, with the others being represented by an “Other” row.

Relative abundances (as %) were also used with the ASV table collapsed to the genus level and visualized as relative abundance bar plots in R [44], with samples grouped by treatment and timepoint. Only the top 10 overall most abundant genera were shown, with the remainder being represented by the “Other” group.

**Alpha diversity analysis**

Alpha diversity was calculated by subsampling the ASV table at 10 different depths, ranging from 380 to 3800 sequences, for the Faith’s Phylogenetic Diversity [45], Observed Features [46], and Pielou’s Evenness [47] metrics. Twenty iterations were performed at each depth to obtain average alpha diversity values. Averages for the greatest depth were used to determine if any of the alpha diversity metrics differed significantly based on treatment and time point (Kruskal-Wallis, P ≤ 0.05). Pre denotes “Baseline” or “Control”.

**Beta diversity analysis**

Beta diversity analyses were conducted after the ASV table had first undergone cumulative sum scaling normalization [48]. Distances between samples were calculated using the Weighted Unifrac metric [49] based on the normalized table and rooted tree. The resulting distance matrix was visualized as a Principal Coordinates Analysis (PCoA) plot. Statistical differences between sample groupings based on treatment and timepoint were evaluated (PERMANOVA, P ≤ 0.05).

**Biomarker analysis**

Biomarker analysis was performed using the linear discriminant analysis effect size (LEfSe) [50] to identify taxa that had significantly different abundances based on timepoint within the same treatment and between different treatments. The ASV table was collapsed to level seven (species) and normalized with the counts per million method. Only taxa identified as having significantly differential abundances (Kruskal-Wallis, P ≤ 0.05) with a log (LDA) score of at least 2.0 were considered to be enriched. This analysis could not be conducted for the ABX treatment group, as only one sample had over 1,000 sequences after quality and decontamination filtering.

**Mouse model of severe sciatic nerve crush injury**

While it is not possible to investigate the mechanisms of poor functional outcomes after traumatic peripheral nerve injury (TPNI) in humans, rodent models of TPNI are widely used to investigate the effect of novel therapeutic strategy [35, 51]. An established severe sciatic nerve crush injury model was utilized as previously described [51, 52]. Briefly, after intraperitoneal (IP) ketamine (100 mg/kg)/xylazine (10 mg/kg) anesthesia, the sciatic nerve was bluntly exposed through the iliotibial band and crush injury (34 MPa, 30s) was performed ~3 mm proximal to the sciatic nerve trifurcation using a needle driver (2 mm tip width; V. Mueller, RH 2560, German Stainless) [52]. The skin was closed by surgical staples and post-operative slow release buprenorphine (0.05 mg/kg) was given subcutaneously to all animals as an analgesic.

**Walking Track Analysis to Determine Sciatic Function Index**

To measure functional recovery following TPNI, walking track analysis was performed to calculate the sciatic function index (SFI) before (baseline) ABX or PBX treatment and at 1, 3, 7, 14 and 21 days post-injury as previously described [51, 53, 54].

**Euthanasia**

Euthanasia of animals was performed as per American Veterinary Medical Association (AVMA) and IACUC guidelines. After final walking track analysis, mice were euthanized under deep anesthesia with ketamine (100 mg/kg)/xylazine (10 mg/kg) mixture followed by cervical dislocation and the death of animal was confirmed by the loss of consciousness and cessation of respiration. The carcasses were disposed at the Animal Care Facility.

**Data analysis.** All results are presented as means ± SEM. Sciatic function index is the main parameter affected by a nerve injury. In our preliminary studies, we calculated the sample size required for each group to reach the 5% significance level through computer simulation. This power analysis showed that at least 5-7 mice in each group are required for nerve crush injury [51, 52]. Functional data were analyzed by a mixed model 2-way ANOVA for multiple comparisons with Tukey’s correction using the GraphPad PRISM 8 (GraphPad Software, San Diego, CA, USA) with *P*<0.05 considered significant. Significant differences for the microbiome alpha diversity between the groups were assessed using Kruskal-Wallis tests through QIIME2. Likewise, beta diversity differences were assessed using PERMANOVA tests through QIIME2. Wilcoxon Rank Sum tests within R were used to test for significant differences among the most abundant phyla, as well as specific taxa of interest (*Akkermansia, Bifidobacteriales, and Lactobacillales*) based on treatment and timepoint. For all tests, P value of <0.05 was considered a statistically significant value.
