# Supplementary figures and images for: Altered gut microbiota composition with antibiotic treatment impairs functional recovery after traumatic peripheral nerve crush injury in mice: effects of probiotics with butyrate producing bacteria
Source: BMC Res Notes. 2022 Feb 23;15:80. doi: 10.1186/s13104-022-05967-8 (PMC8867741; doi:10.1186/s13104-022-05967-8)

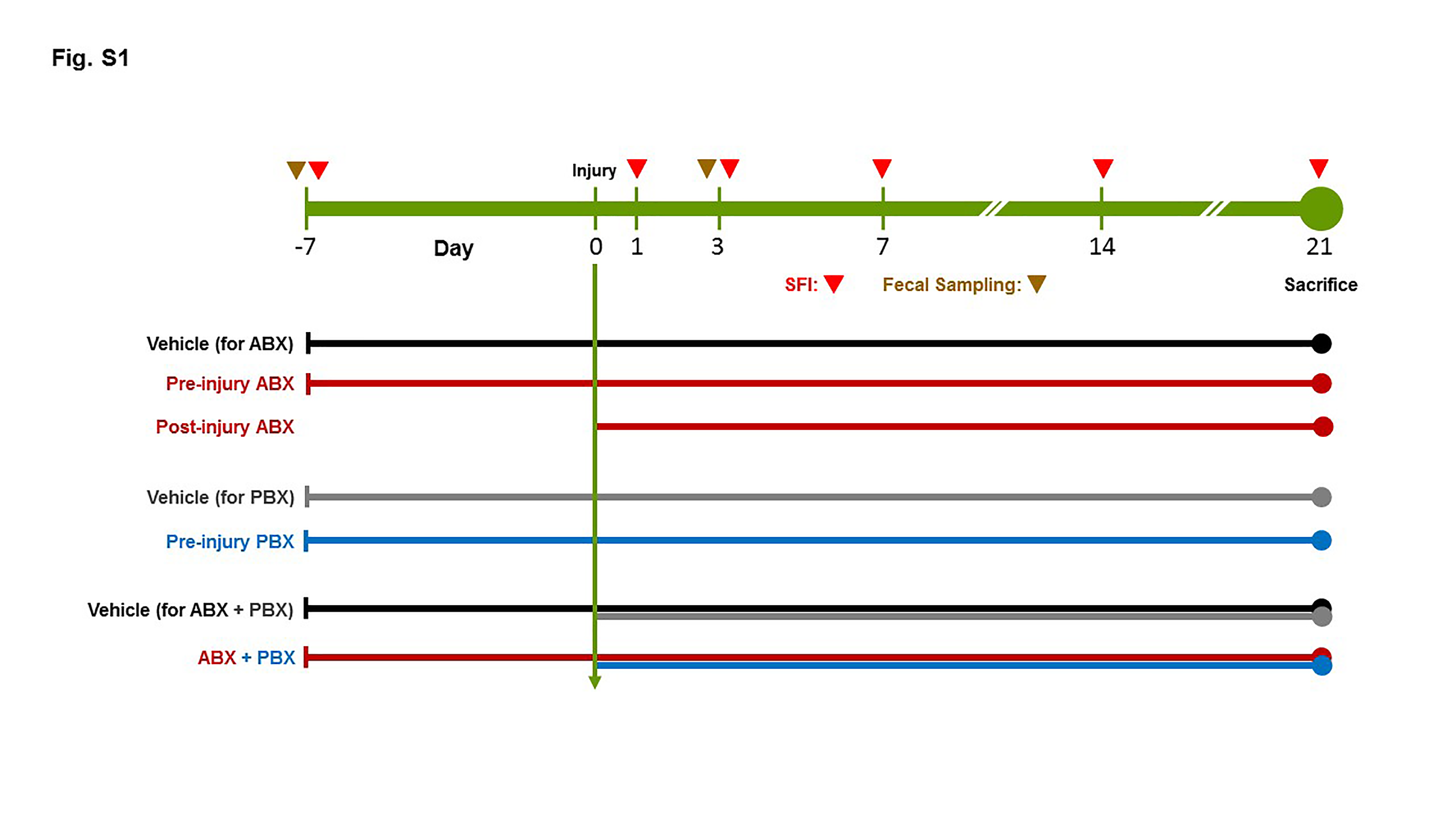

Supplement: Supplementary file 2 — Additional file 2: Figure S1. Experimental groups and time lines. Vehicle for ABX group received autoclaved drinking water supplemented with maple syrup daily beginning 7 days before nerve injury (Day − 7); Pre-injury ABX group received the antibiotic cocktail in drinking water daily beginning 7 days before nerve injury (Day − 7); Post-injury ABX group received the antibiotic cocktail in drinking water daily immediately after nerve injury (Day 0); Vehicle for PBX group received 400 µL sterile saline via oral gavage daily beginning 7 days before nerve injury (Day − 7); Pre-injury PBX group received probiotics suspension via oral gavage daily beginning 7 days before nerve injury (Day − 7); Vehicle for ABX and PBX group received autoclaved drinking water supplemented with maple syrup daily beginning 7 days before nerve injury (Day − 7) plus 400 µL sterile saline via oral gavage daily immediately after nerve injury (Day 0); and sequential ABX + PBX group received the antibiotic cocktail in drinking water daily beginning 7 days before nerve injury (Day − 7) plus probiotics suspension via oral gavage immediately after nerve injury (Day 0). All groups received a similar crush injury and each treatment regimen was continued daily until the end of protocol at day 21. Functional analysis as sciatic function index (SFI) and fecal sampling were performed at indicated days. [file 13104_2022_5967_MOESM2_ESM.tif]

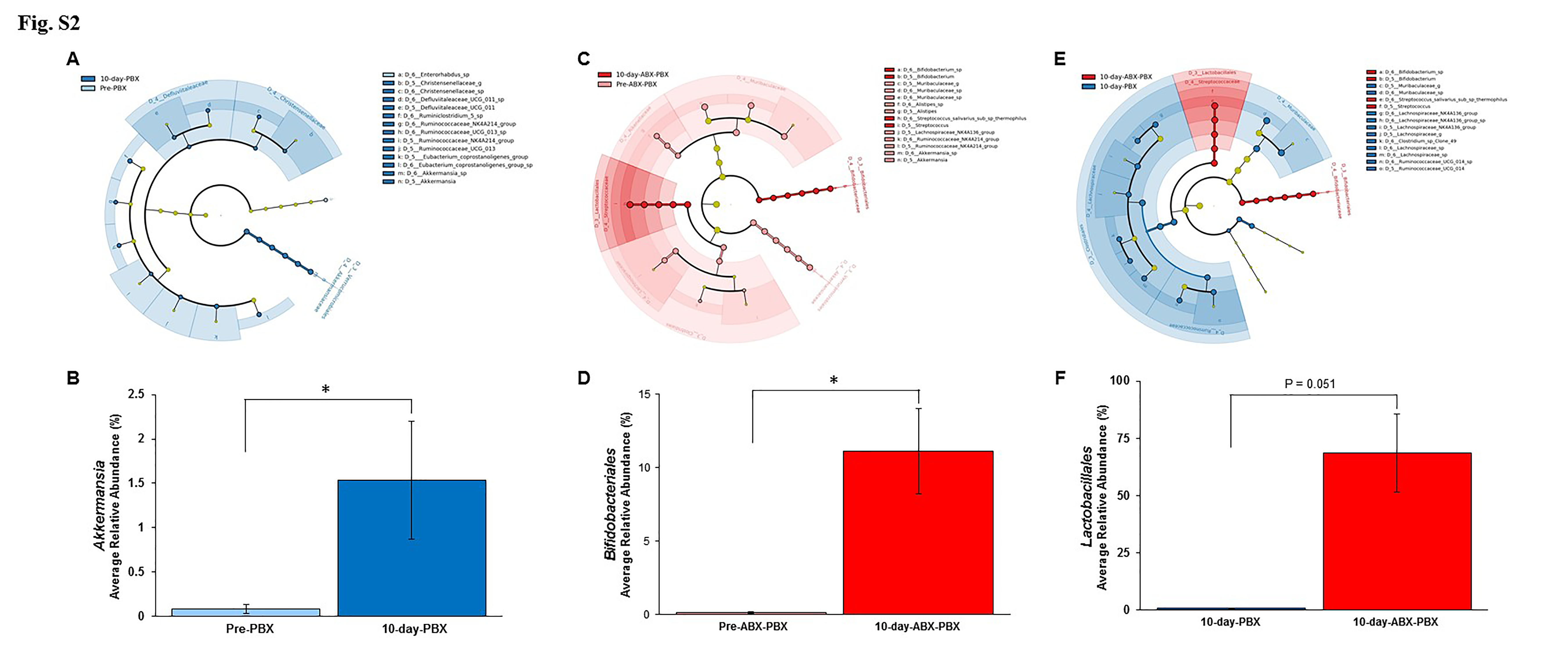

Supplement: Supplementary file 4 — Additional file 4: Figure S2. Taxonomic differences of fecal microbiota between different groups of mice. A Cladogram using LEfSe method showing the phylogenetic relationships among the enriched taxa within Pre-PBX (light blue) and 10-day-PBX groups (blue). B The relative abundance of Akkermansia was significantly higher in 10-day-PBX group. *P < 0.05, n = 6/group. C Cladogram using LEfSe method showing the phylogenetic relationships among the enriched taxa within Pre-ABX-PBX (pink) and 10-day-ABX-PBX (red) groups. D The relative abundance of Bifidobacteriales was significantly higher in 10-day-ABX-PBX group. *P < 0.05, n = 5–6/group. E Cladogram using LEfSe method showing the phylogenetic relationships among the enriched taxa within 10-day-PBX (blue) and 10-day-ABX-PBX (red) groups. F The relative abundance of Lactobacillales was markedly higher in 10-day-ABX-PBX group. P = 0.051, n = 5–6/group. [file 13104_2022_5967_MOESM4_ESM.tif]
