# Supplementary material for: Altered gut microbiota composition with antibiotic treatment impairs functional recovery after traumatic peripheral nerve crush injury in mice: effects of probiotics with butyrate producing bacteria
Source: BMC Res Notes. 2022 Feb 23;15:80. doi: 10.1186/s13104-022-05967-8 (PMC8867741; doi:10.1186/s13104-022-05967-8)
Supplement: Supplementary file 3 — Additional file 3: Table S1. The top characterized taxa at the phylum level of each group before (Pre-) treatment and at 10-day of the study. [file 13104_2022_5967_MOESM3_ESM.docx]

## Table S1. 16s rRNA gene sequencing analysis of stool showing the top six characteristic taxa at the phylum level of each group before treatment (Pre-) and at 10-day of the study. The 10-day of the study means treatment started 7 days before nerve injury and continued for 3 days after the injury, that is day 3 as shown in Additional file 2- Fig. S1. Maple syrup (MS), antibiotics (ABX), probiotics (PBX), and antibiotics plus probiotics (ABX-PBX). n, number of animals used for stool collections.

| **Phyla (%)** | **Pre-MS** | **10-day-MS** | **Pre-ABX** | **10-Day-ABX** | **Pre-PBX** | **10-Day-PBX** | **Pre-ABX-PBX** | **10-Day-ABX-PBX** |
| --- | --- | --- | --- | --- | --- | --- | --- | --- |
| **Actinobacteria** | 0.01±0.01 | 0.02±0.02 | 0.02±0.02 | 6.66 | 0.02±0.02 | 0.29±0.47 | 0.22±0.23 | 11.15±6.51 |
| **Bacteroidetes** | 48.21±27.4 | 41.37±12.95 | 74.12±10.32 | 2.15 | 65.17±20.72 | 57.70±18.4 | 66.67±13.61 | 20.09±44.31 |
| **Firmicutes** | 49.86±28.05 | 54.07±11.27 | 20.89±10.63 | 85.01 | 33.38±19.72 | 38.37±18.36 | 14.38±7.98 | 68.73±38.27 |
| **Proteobacteria** | 0.49±0.36 | 0.39±0.14 | 0.69±0.26 | 5.36 | 0.14±0.06 | 0.10±0.06 | 0.27±0.29 | 0.02±0.03 |
| **Tenericutes** | 1.32±1.1 | 1.34±0.77 | 2.15±1.31 |  | 1.20±1.24 | 2.00±1.71 | 0.39±0.31 |  |
| **Verrucomicrobia** | 0.11±0.1 | 2.80±3.54 | 2.12±2.18 | 0.33 | 0.08±0.13 | 1.54±1.62 | 18.07±10.72 |  |
| **Others** | 0.00±0 | 0.00±0 | 0.10±0 | 0.10±0 | 0.00±0 | 0.00±0 | 0.00±0 | 0±0.01 |
| **n** | 7 | 7 | 6 | 1 | 6 | 6 | 6 | 6 |
